# Antinociceptive effect of intrathecal injection of miR-9-5p modified mouse bone marrow mesenchymal stem cells on a mouse model of bone cancer pain

**DOI:** 10.1186/s12974-020-01765-w

**Published:** 2020-03-16

**Authors:** Chao Zhu, Kun Wang, Zhi Chen, Yingchao Han, Hao Chen, Quan Li, Zude Liu, Lie Qian, Jun Tang, Hongxing Shen

**Affiliations:** 1 Department of Spine Surgery, Renji Hospital, School of Medicine, Shanghai Jiao Tong University, Shanghai, 200240 China; 2 Department of Orthopaedics, 987 Hospital of PLA, Xi’an, 721000 Shaanxi Province China; 3 Department of Anesthesiology, Jinling Hospital, Medical School of Nanjing University, No. 305 East Zhongshan Road, Nanjing, 210002 Jiangsu China

**Keywords:** BMSC, Bone cancer pain, Cytokine, REST, MOR

## Abstract

**Background:**

A growing body of studies have indicated that bone marrow mesenchymal stem cells (BMSCs) have powerful analgesic effects in animal models of bone cancer pain. Here, we explored the molecular mechanisms underlying how BMSCs alleviate pain sensation in a mouse model of bone cancer pain.

**Methods:**

C3H/HeN adult male mice were used to generate a bone cancer pain model. BMSCs were isolated from mouse bone marrow, modified by transfection with microRNA-9-5p (miR-9-5p), and infused into the spinal cord. Spontaneous flinches, paw withdrawal latency, limb-use score, and weight-bearing score were used to assess pain-related behaviors. ELISA, RT-PCR, western blot, and luciferase assay were used to assess gene expressions.

**Results:**

Our results show that miR-9-5p regulated the expression of both repressor element silencing transcription factor (REST) and μ-opioid receptors (MOR) by targeting REST in primary mouse BMSCs. Overexpression of miR-9-5p reversed the activation of inflammatory pathway in TNF-α- and IL-6-treated BMSCs. In addition, miR-9-5p modified BMSCs alleviated cancer pain in the sarcoma-inoculated mouse model. MiR-9-5p modified BMSCs suppressed cytokine expression in the spinal cord of sarcoma-inoculated mice by suppressing REST gene expression.

**Conclusions:**

Our results indicate that miR-9-5p modified BMSCs can relieve bone cancer pain via modulating neuroinflammation in the central nervous system, suggesting genetically modified BMSCs could be a promising cell therapy in pain management.

## Introduction

Among cancer patients, 70% report troublesome cancer-related pain attributable to poor quality of life [1, 2]. Worldwide, up to 50% patients with cancer-related pain symptoms are inadequately controlled. Among cancer-related pain, bone cancer pain is the most severe and devastating one [3]. Cancers including the prostate, kidney, breast, and lung commonly metastasize to bones such as the tibia hip and femur. The bone cancer pain can be ongoing pain which usually has a dull feature or breakthrough pain which is severe spontaneous pain with unpredictable and acute occurrence. The breakthrough pain is usually caused by bone remodeling process and can significantly disturb patient’s functional status. Recently, improving quality of pain management has become one of the focus in clinical practice [4, 5]. Understanding the underlying molecular and cellular mechanisms of bone cancer pain will help to discover more effective treatment with less side effect profile.

Over the past decades, growing efforts have been committed to uncover the cellular and molecular mechanisms underlying pain sensation. The efforts in exploring treatment of cancer-related have shifted beyond traditional medicine to novel biological approaches, such as stem cell transplantation. Recently, bone marrow mesenchymal stem cells (BMSCs) turn out to be the most promising cell therapy attributable to their well-known properties including differentiation into neural cells, migration to sites of injury, and immunosuppressive features [6]. More importantly, BMSCs can be genetically manipulated and thus have the capacity to accommodate substantial bioactivities [7]. Studies have shown that genetically modified BMSCs can significantly inhibit pain sensation [8]. However, the exact molecular mechanisms underlying BMSC effect on pain remain poorly understood.

Opioids and its receptors are the key entry point for the development of pain relievers as well as underpinning the pathways related to pain sensation [9]. Nowadays, opioids remain to be the most frequently prescribed medication for pain management in patients with bone cancer pain [10]. It has been reported that the expression and modulation of μ-opioid receptors (MOR) regulated by different molecular pathways such as cytokine signaling pathways and a variety of transcriptional factors contribute to nociceptive behavior abnormalities related to bone cancer pain [11, 12]. However, the exact molecular mechanisms underlying regulation of MOR expression remains poorly understood. Repressor element silencing transcription factor (REST) together with repressor element 1 (NF1) is involved in regulating a large number of neuron-specific gene expression in the central nervous system. MOR is one of the neuronal genes regulated by REST. In vitro, REST regulates MOR expression by directly inhibiting MOR transcription [13, 14]. In vivo, it is found that REST can modulate MOR gene expression profile by silencing MOR gene promoter epigenetically [15]. Interestingly, REST is also regulated by a variety of cytokines that are known to trigger pain sensation in different pathological situations [16]. In situation of extreme pain like bone cancer pain, little is known regarding how the crosstalk among REST, MOR, and cytokines is formed in the cellular pathway network. MicroRNAs have been shown to be the major gene expression regulators involved in multiple cellular processes [17]. In the present study, we hypothesize that microRNA-9-5p might be the bridge connecting the regulation of REST, MOR, and cytokine expressions in the BMSCs. Our results demonstrate that the crosstalk among NEST, MOR, cytokines, and microRNA-5-9P in the transplanted BMSCs eventually contribute to the pain alleviation mediated by BMSCs.

## Methods and animals

### Experimental animals

Twenty-five- to 30-g C3H/HeN adult male mice were used in the present study. The mice housing and maintenance were as described previously [15]. Specifically, the mice were attained from Weitong Lihua Laboratory Animal Technology Co, Ltd., Beijing, China. All animal-related procedures complied with the NIH guidance for laboratory animals as well as the ISAP ethical issues. All experimental approaches were reviewed and approved by the Animal Use and Care Committee in Renji Hospital, School of Medicine, Shanghai Jiao Tong University.

The mouse bone cancer pain model was generated as previously described [15]. Specifically, the NCTC murine sarcoma cells were purchased from American Type Culture Collection, Rockville, MD. Horse sera were from HyClone, Logan, UT. Sham mice were generated using the same surgical procedures, and only 25-ml medium was injected into the distal femur condyle. The surgery was conducted 5 days after catheter implantation which was for BMSC infusion.

### Mouse BMSC isolation, culture, and characterization

The BMSCs were isolated and cultured following the published protocol by Soleimani and Nadri [18]. Particularly, in the present study, the bone marrow was flushed out of the hind limbs of C3H/HeN adult male mice 5–6-weeks old using Dulbecco’s modified Eagle’s medium (DMEM) and bone marrow cells were cultured in a density of 25 × 10^6^ cells/ml. The fetal bovine serum was purchased from HyClone, GE Healthcare Life Sciences, Logan, UT, USA. Penicillin and streptomycin were from Beyotime Institute of Biotechnology, Haimen, China. Phycoerythrin-marked rabbit anti-mouse CD29 or CD34 (Serotec, Ltd., UK) and fluorescein isothiocyanate-linked anti-mouse CD44 or CD45 antibody (Serotec, Ltd., UK) were used to stain cell surface markers which subsequently were evaluated using fluorescence-activated cell sorting with flow cytometry. Oil Red O was used to assess cell capability of adipogenesis. The capability of osteogenesis was evaluated using calcium tubercle sodium alizarinsulfonate staining.

### Mouse BMSC infusion

Catheters were surgically implanted into C3H/HeN adult male mice by laminectomy at L3-L4 spinal level. A 25-gauge needle and a catheter were inserted into the subdural area. The proximal part of the catheter was sutured into the subcutaneous tissue to secure the catheter from being removed. Mice were grouped into four categories: control group, sham group, bone cancer pain group, and bone cancer pains + BMSC group which received 6 × 10^6^ cells/10 μL BMSC infusion every day started 2 days before murine sarcoma cell implantation till 21 post operation.

### Behavioral assessment

Behavioral assessment related to pain sensation was as described previously [15]. In the present study, spontaneous flinches were used to assess the ongoing pain and paw withdrawal latency (PWT) was used to assess mechanical allodynia. All mice received three assessments in an environment where they had been habituated for at least 30 min before all experimental procedures were carried out and the average score was regarded as the baseline value. On days 3, 7, 14, and 21 post operation, all mice received the same assessments conducted by the same experimenter who are blinded to the treatment regimen. For spontaneous flinches, the mice were observed for 2 min and a scale as described previously [15] was adopted for scoring flinching behaviors. For PWT, von Frey filaments with a weight range as described in our previous study [15] were used to stimulate the hind paws and every mouse was tested for 5 times per stimulus. The threshold was defined as the lowest von Frey filament and strength inducing equal to or more than three positive responses.

### Lentiviral vector production and intrathecal injection

Lentiviral vectors including LV-miR-9-5p mimics, LV-REST, and their LV-NC were generated based on the instructions of the manufacturer (RiboBio, Guangzhou, China). Bilateral hind limbs were paralyzed by injecting lidocaine (Sigma, St Louis, MO). Then, the intrathecal implantation was performed by inserting a PE-10 polyethylene catheter into the cisterna magna. For each intrathecal administration, recombinant lentivirus (10 μL, RiboBio, Guangzhou, China) was injected into the intrathecal catheter by using a microinjection syringe.

### Cell transfection and treatment

The BMSC cells were seeded in 6-well plates at a density of 1 × 10^5^ per well and transfected with lentiviral vectors. Approximately 18 h later, cells were treated with TNF-α at a concentration of 25 ng/mL (Roche Molecular Biochemicals, Mannheim, Germany) and IL-6 at a concentration of 0.1 ng/mL (Sigma). Seventy-two hours after treatment, cells were collected for subsequent experiments.

### qRT-PCR

RNAeasy mini kit (Cat No.74104, Qiagen, USA) was used to extract total RNA. The Prime Script RT Master Mix was then used to reverse transcribe RNA into cDNA. SYBR Premix Ex Taq II and the Applied Biosystems 7900 Real-Time PCR System (Applied Biosystems, Foster City, CA) were used for amplification and quantification of cDNA (TaKaRaBio Technology). Primers are listed in Table 1.

**Table 1:** List of primers for qRT-PCR

IL-6	Forward	5′-TCCAGTTGCCTTCTTGGGAC-3′
	Reverse	5′-GTGTAATTAAGCCTCCGACTTG-3′
TNF-α	Forward	5′-CATCTTCTCAAAATTCGAGTGACAA-3′
	Reverse	5′-TGGGAGTAGACAAGGTACAACCC-3′
IL-1β	Forward	5′-TTTTCCTCCTTGCCTCTGAT-3′
	Reverse	5′-GAGTGCTGCCTAATGTCCCC-3′
GAPDH	Forward	5′-TTCACCACCATGGAGAAGGC-3′
	Reverse	5′-GGCATGGACTGTGGTCATGA-3′
Caspase-3	Forward	5′-GGTGTTGATGATGACATGGCG-3′
	Reverse	5′-GTACCTCTGCAGCATGAGAGTAG-3′
miR-9-5p	Forward	5′-GGACGGACAGCGAGAGGAGGCCAAA-3′
	Reverse	5′-TTTGGCCTCCTCTCGCTGTCCGTCC-3′
MOR	Forward	5′-ATCCTCTCTTCTGCCATTGGT-3′
	Reverse	5′-TGAAGGCGAAGATGAAGACA-3′
REST	Forward	5′-GTGCGAACTCACACAGGAGA-3′
	Reverse	5′-AAGAGGTTTAGGCCCGTTGT-3′

### Enzyme-linked immunosorbent assay

On day 21 post operation, the mice were sacrificed and the tissues of the dorsal spinal cord were treated with a lysis buffer and phenylmethanesulfonyl fluoride, incubated in a freezer for 10 min, and then centrifuged for 15 min at 4 °C at a speed of 10,000*g*. Supernatants were then collected to test the protein expression of IL-1beta, IL-6, and TNF-α using enzyme-linked immunosorbent assay (ELISA) Kits. All experiments followed the manufacturers’ instructions. Specifically, TNF-α kit was purchased from Jingkang Biological Engineering Co., Ltd., China; IL-1beta kit was from Tiderad Biotechnology, China; and IL-6 kit was from Dingguo Changsheng Biotechnology, China. A microplate reader (Bio-Rad, Hercules, CA) was used to examine the optical density at A450.

### Western blotting analysis

Western blotting and quantitative analysis were performed as described previously [15]. Specifically, primary rabbit anti-mouse MOR antibody was obtained from MYBiosource, LLC, USA (catalog #MBS5300361), and primary rabbit anti-mouse REST antibody was obtained from Sigma, USA (catalog #07-579). Goat secondary antibody conjugated with horseradish peroxidase was used at 1:5000 dilution (P0008, Promoter, Wuhan, China). An ECL-based system (Santa Cruz Biotechnol., CA, USA) was used for imaging and quantification. GAPDH was used as an internal reference.

### Luciferase reporter assay

**Fig. 1:**
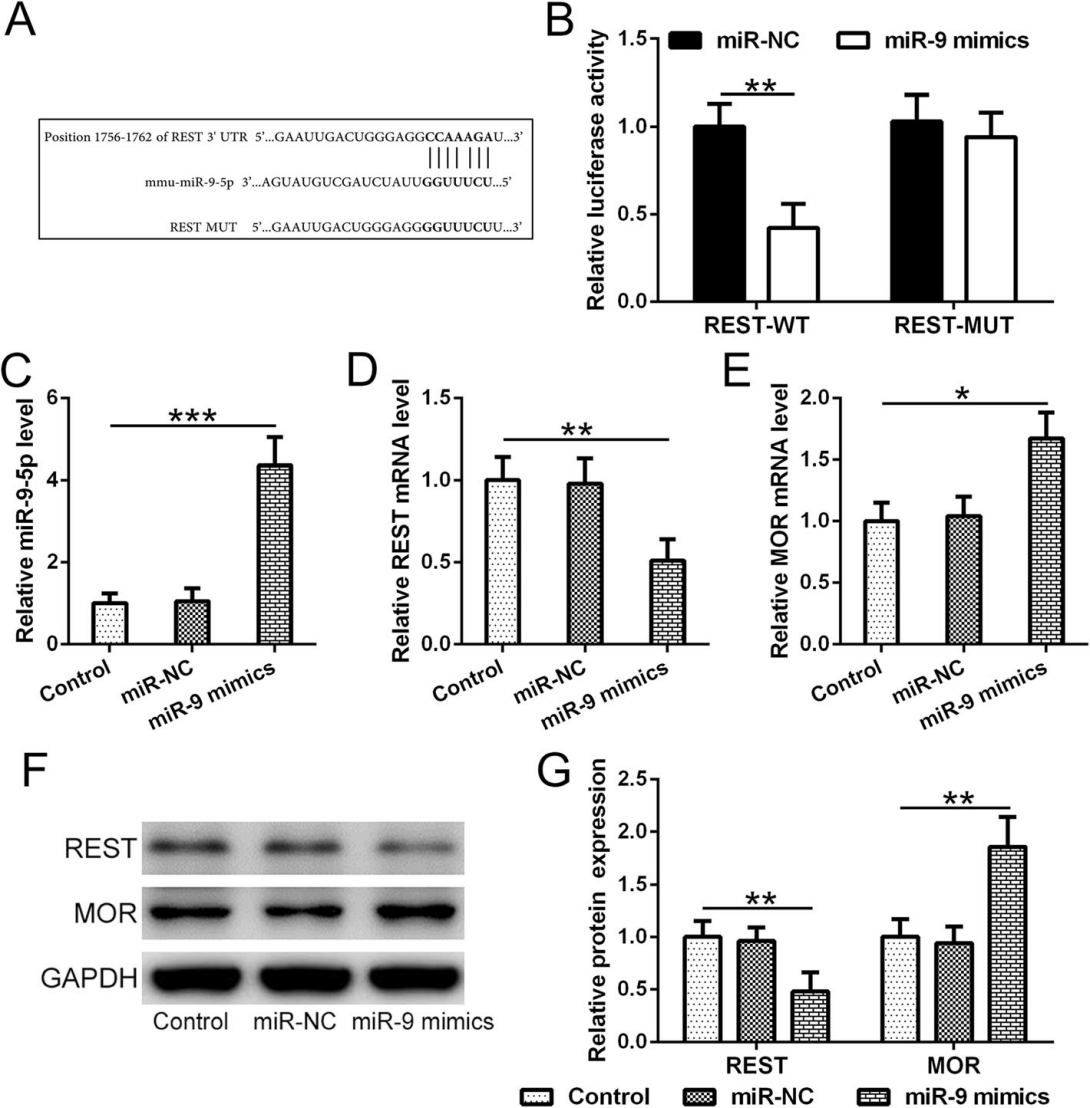
miR-9-5p regulated expression of both REST and MOR by targeting REST in primary mouse bone marrow mesenchymal stem cells (BMSCs). **a** DNA sequence of a predicted binding site of mmu-miR-9-5p within the wild-type 3′-UTR region of REST mRNA as well as a mutated 3′-UTR of REST was cloned into a luciferase reporter construct, respectively. The corresponding mRNA sequence was shown. **b** miR-9-5p suppressed the luciferase activities expressed by WT-REST but not MUT-REST luciferase construct. Luciferase activities were measured at 48 h post transfection. **c** Expression efficacy of miR-9-5p mimics was verified using qRT-PCR at 24-h post transfection. **d**, **e** Overexpression of miR-9-5p decreased REST but increased MOR mRNA levels 24 h post transfection. **f** Accordingly, overexpression of miR-9-5p decreased REST but increased MOR protein expression 24 h post transfection. **g** Quantification of protein expression presented in **f**. qRT-PCR was used to evaluate mRNA level, and western blotting was used to evaluate protein expression. Mean ± SD is used to present all data. *p < 0.05, **p < 0.01, and ***p < 0.001

The sequence of WT or MUT miR-29-5p binding site at 3′-UTR of REST1 is shown in Fig. 1. The corresponding oligo DNA fragment was cloned into pGL3 basic vector (Promega) and transfected into HEK 293T cells together with miR-29-5p mimic construct by using lipofectamine following the manufacturer’s instruction (RiboBio, Guangzhou). The luciferase activity was evaluated using a Dual-Luciferase Reporter Assay System (Promega, Madison, WI) 24 h later.

### Statistical analysis

SPSS (V16, SPSS Inc., Chicago, IL) statistical analyses were used to collect and analyze data. All data are presented as the means ± SD. One- or two-way ANOVA analysis followed by Bonferroni correction was conducted. Significance was considered when *p* has a value less than 5%.

## Results

### MiR-9-5p regulated expression of both REST and MOR by targeting REST in primary mouse BMSCs

To verify whether REST is a potential target of miR-9-5P, we performed a sequence alignment analysis first between sequences of miR-9-5p RNA and REST mRNA 3′-UTR. A predicted binding site of mmu-miR-9-5p within the wild-type 3′-UTR region of REST mRNA was identified (Fig. 1a). To test whether this predicted miR-9-5p binding site can effectively regulate REST expression, we further designed a mutated 3′-UTR of REST and then cloned the wide type or mutated REST 3′-UTR into a luciferase reporter construct, respectively (Fig. 1a). As expected, miR-9-5p suppressed the luciferase activities expressed by WT-REST but not MUT-REST luciferase construct (Fig. 1b). To rule out the suppression of luciferase activities is an artifact from transfection, we performed qRT-PCR to test the expression efficacy of miR-9-5p mimics (Fig. 1c). As shown in Fig. 1c, miR-9-5p mRNA levels substantially increased in cells transfected with miR-9-5p mimics. We then tested whether miR-9-5p mimics could suppress REST mRNA and protein levels. As shown in Fig. 1d–g, overexpression of miR-9-5p reduced REST mRNA levels as well as protein expression 24 h post transfection. Since REST has been reported to modulate MOR expression, we hypothesize that a decrease in REST expression probably leads to modulation of MOR expression. Our results indicated that MOR mRNA level and protein expression increased in BMSCs overexpressing miR-9-5p (Fig. 1f, g). To find out whether changes in REST and MOR expression pattern will change the cellular properties of BMSCs, we checked the cell surface markers for stem cells. Our results showed that the stem cell properties were well preserve after miR-9-5p mimic transfection (Table 2). Taken together, our results indicate that miR-9-5p modulates expressions of REST and MOR, suggesting miR-9-5p might play a key role in pain sensation.

**Table 2:** Analysis of cell surface marker expressions by flow cytometry after transfection (%, mean ± SD)

Groups (BMSCs)	Number	CD29	CD44	CD34	CD45
Control	4	99.04 ± 0.68	94.74 ± 1.74	0.35 ± 0.16	1.75 ± 0.46
miR-NC	4	98.46 ± 0.83	95.01 ± 2.42	0.41 ± 0.31	1.52 ± 0.41
miR-9 mimics	4	98.79 ± 1.06	93.68 ± 2.68	0.54 ± 0.35	1.69 ± 0.51
*p* value	–	0.214	0.124	0.367	0.395

BMSCs among different groups were analyzed for CD 29, 34, 44, and 45 expressions using fluorescence-activated cell sorting (FACS) with flow cytometry at passage 3. The lack of CD 34 and 45 expressions and the presence of CD 29 and 44 expressions indicated a remaining mesenchymal stem cell lineage after miR-9 mimic transfection

### Overexpression of miR-9-5p reversed the activation of inflammatory pathway in TNF-α- and IL-6-treated BMSCs

**Fig. 2:**
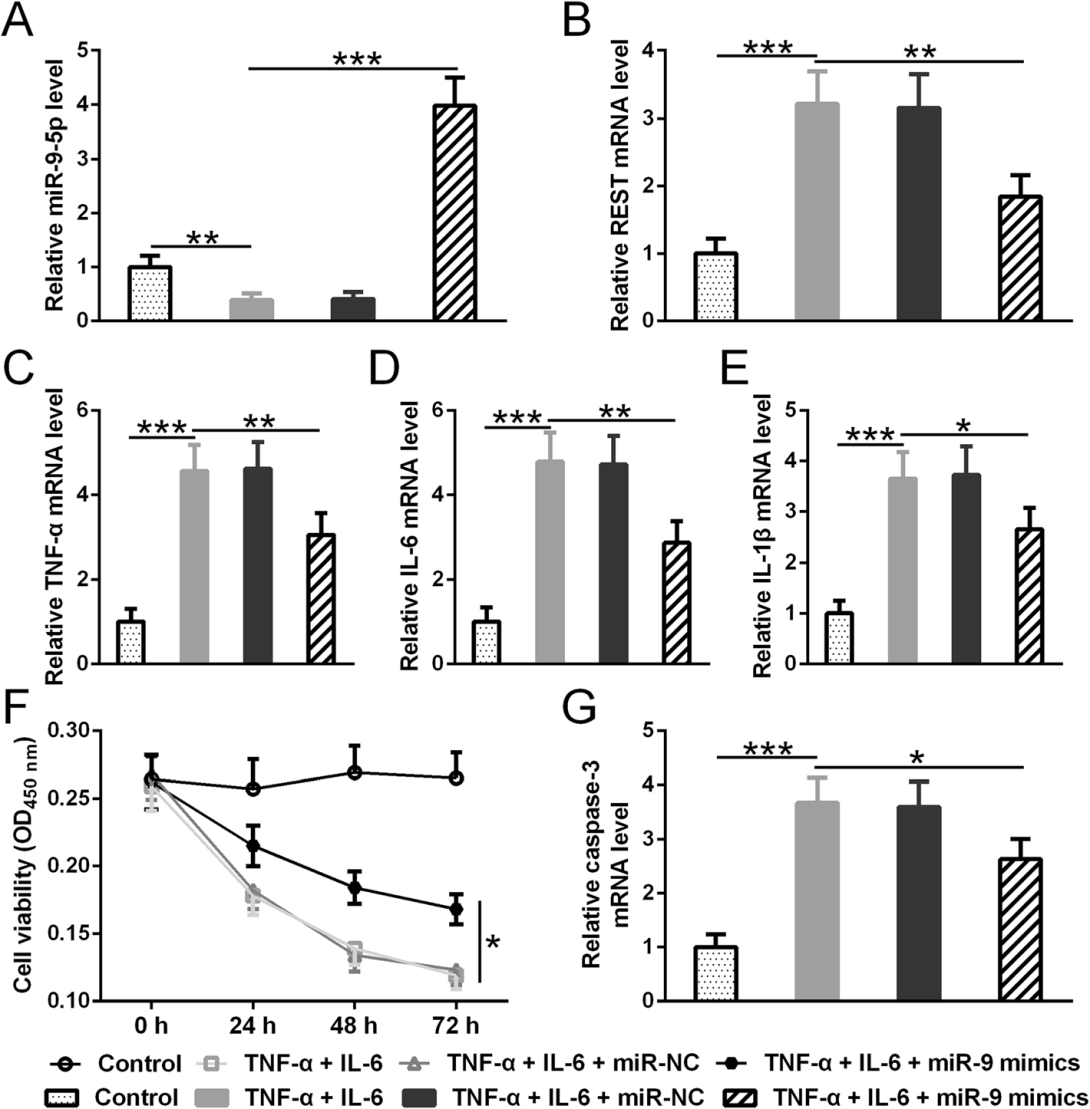
Overexpression of miR-9-5p reversed the activation of inflammatory pathway in TNF-α- and IL-6beta-treated BMSCs. **a** Expression of miR-9-5p was suppressed by IL-6 and TNF-α treatment which was reversed by miR-9-5p mimic transfection. **b** IL-6 and TNF-α treatment enhanced REST mRNA level which was reversed by miR-9-5p mimic transfection. **c**–**e** IL-6 and TNF-α treatment enhanced TNF-α (**c**), IL-6 (**d**), and IL-1beta mRNA which was reversed by miR-9-5p mimic transfection. **f** IL-6 and TNF-α treatment decreases cell viability which was reversed by miR-9-5p mimic transfection. **g** IL-6 and TNF-α treatment enhanced caspase 3 mRNA level which was reversed by miR-9-5p mimic transfection. qRT-PCR was used to evaluate mRNA level. Mean ± SD is used to present all data. *p < 0.05, **p < 0.01, and ***p < 0.001

Cytokines such as TNF-α and IL-6 can enhance pain sensation by modulating central sensitivity in the nervous system and promoting positive feedbacks. To test whether miR-9-5p is involved in the TNF-α and IL-6 signaling pathway, we treated BMSCs with TNF-α and IL-6. Interestingly, IL-6 and TNF-α treatment suppressed endogenous miR-9-5p expression which could be compensated with miR-9-5p mimic transfection (Fig. 2a). In addition, IL-6 and TNF-α treatment enhanced REST mRNA level in BMSCs. Consistent with our previous findings that REST is one of the miR-9-5p target genes, overexpression of miR-9-5p reversed the increase of REST expression which resulted from cytokine treatment (Fig. 2b). Since REST is one of the key transcriptional factors regulating cytokine gene’s transcription, it is not a surprise that an increase of REST expression in TNF-α- and IL-6beta-treated BMSCs led to an increase of IL-6, TNF-α, and IL-1beta mRNA level. These positive feedbacks were interrupted by miR-9-5p overexpression (Fig. 2c–e). Furthermore, miR-9-5p overexpression suppressed IL-6- and TNF-α-induced cell apoptosis by inhibiting caspase 3 expression (Fig. 2f, g). Therefore, our results indicate that miR-9-5p can modify BMSCs by modulating the cytokine mediating signaling pathways which involve REST.

### MiR-9-5p modified BMSCs alleviated cancer pain in the sarcoma-inoculated mouse model

**Fig. 3:**
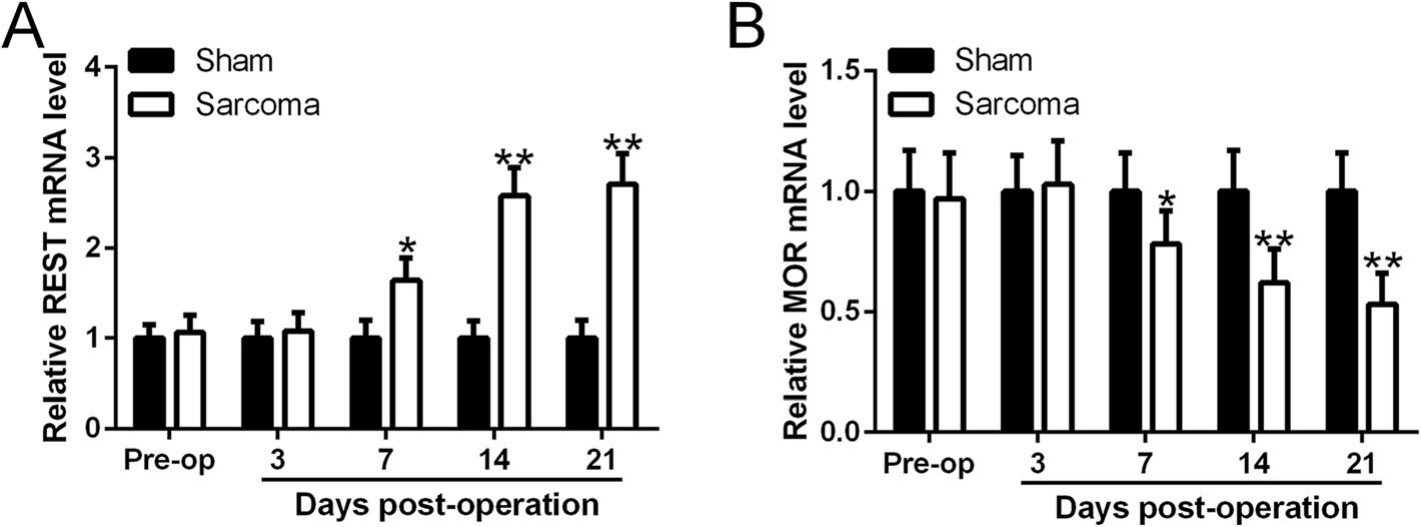
Sarcoma inoculation enhanced REST mRNA level in mice spinal cord (**a**) but suppressed MOR (**b**) mRNA levels in ipsilateral dorsal root ganglion (DRG) of sarcoma-inoculated mice. qRT-PCR was used to measure the mRNA level at different time points post operation. In each group, *n* = 6. Mean ± SD is used to present all data. *p < 0.05, **p < 0.01

**Fig. 4:**
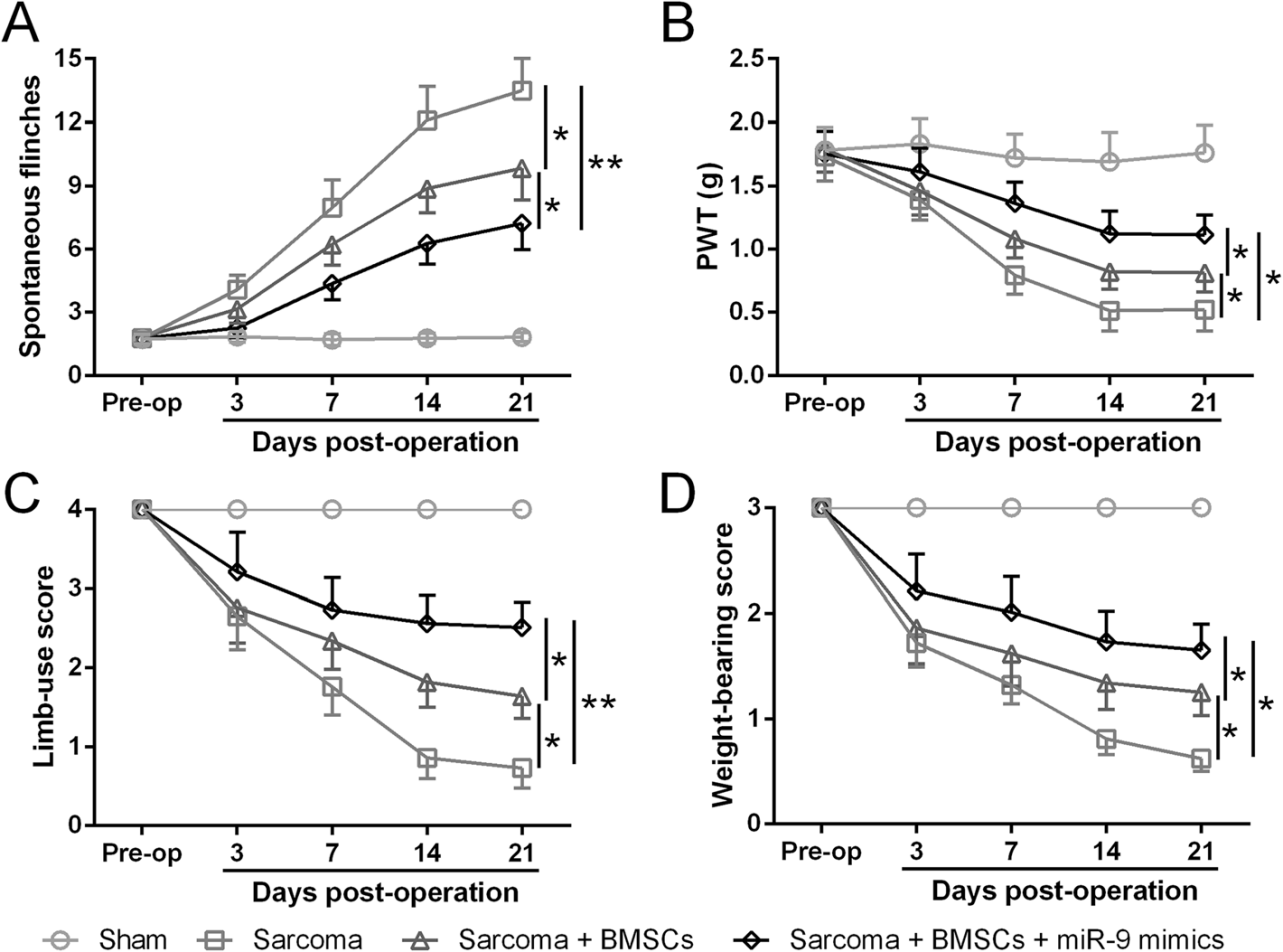
BMSCs overexpressing MiR-9-5p can further alleviate pain-related behaviors in sarcoma-inoculated mice **a**, paw withdrawal threshold **b**, limb-use score **c**, and weight-bearing score **d** were assessed before operation (pre-op) and 3, 7, 14, and 21 days post sarcoma cell inoculation. In each group, *n* = 6. Mean ± SD is presented for data. *p < 0.05, **p < 0.01

To investigate whether miR-9-5p modified BMSCs can alleviate cancer pain, we first checked the expression level of REST as well as MOR whose expression has been reported to be closely associated with REST expression in the mouse sarcoma model. Our results showed that in sarcoma inoculation mouse, the expression of REST mRNA significantly increased while the expression of MOR mRNA levels significantly decreased, suggesting the dysregulation of REST and MOR expression be implicated in the pain sensation caused by sarcoma (Fig. 3a, b). As our previous results showed that miR-9-5p suppressed cytokine expression by targeting REST gene in BMSCs, we hypothesize here that miR-9-5p modified BMSCs can alleviate cancer pain. As expected, transfusion of miR-9-5p modified BMSCs into the spinal cord of sarcoma inoculation mouse model significantly alleviated pain-related behaviors (Fig. 4a–d), including spontaneous flinches, PWT, limb-use score, and weight-bearing score.

### MiR-9-5p modified BMSCs suppressed cytokine expression in the spinal cord of sarcoma-inoculated mice by suppressing REST gene expression

**Fig. 5:**
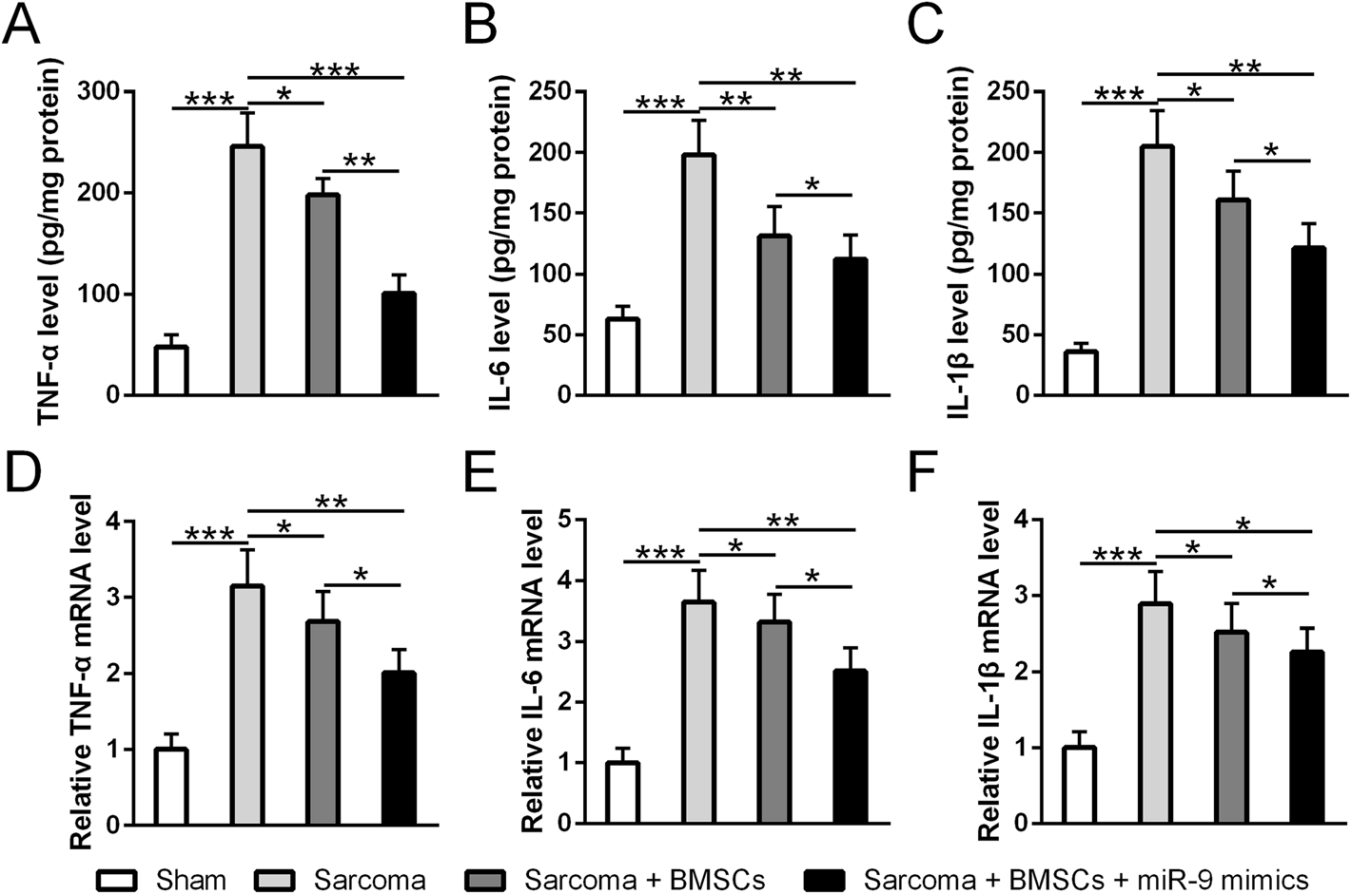
MiR-9-5p modified BMSCs further inhibited inflammatory response in sarcoma-inoculated mice. **a**–**c** MiR-9-5p promoted BMSCs’ capability of suppressing TNF-α (**a**), IL-6 (**b**), and IL-1β (**c**) release in ipsilateral dorsal root ganglion at postoperative day 21. **d**–**f** MiR-9-5p promoted BMSCs’ capability of suppressing TNF-α (**d**), IL-6 (**e**), and IL-1β (**f**) mRNA level in ipsilateral dorsal root ganglion at postoperative day 21. qRT-PCR was used to measure the mRNA level. ELISA was used to measure cytokine concentration. In each group, *n* = 6. Mean ± SD data is used to present all data. *p < 0.05, **p < 0.01, and ***p < 0.001

**Fig. 6:**
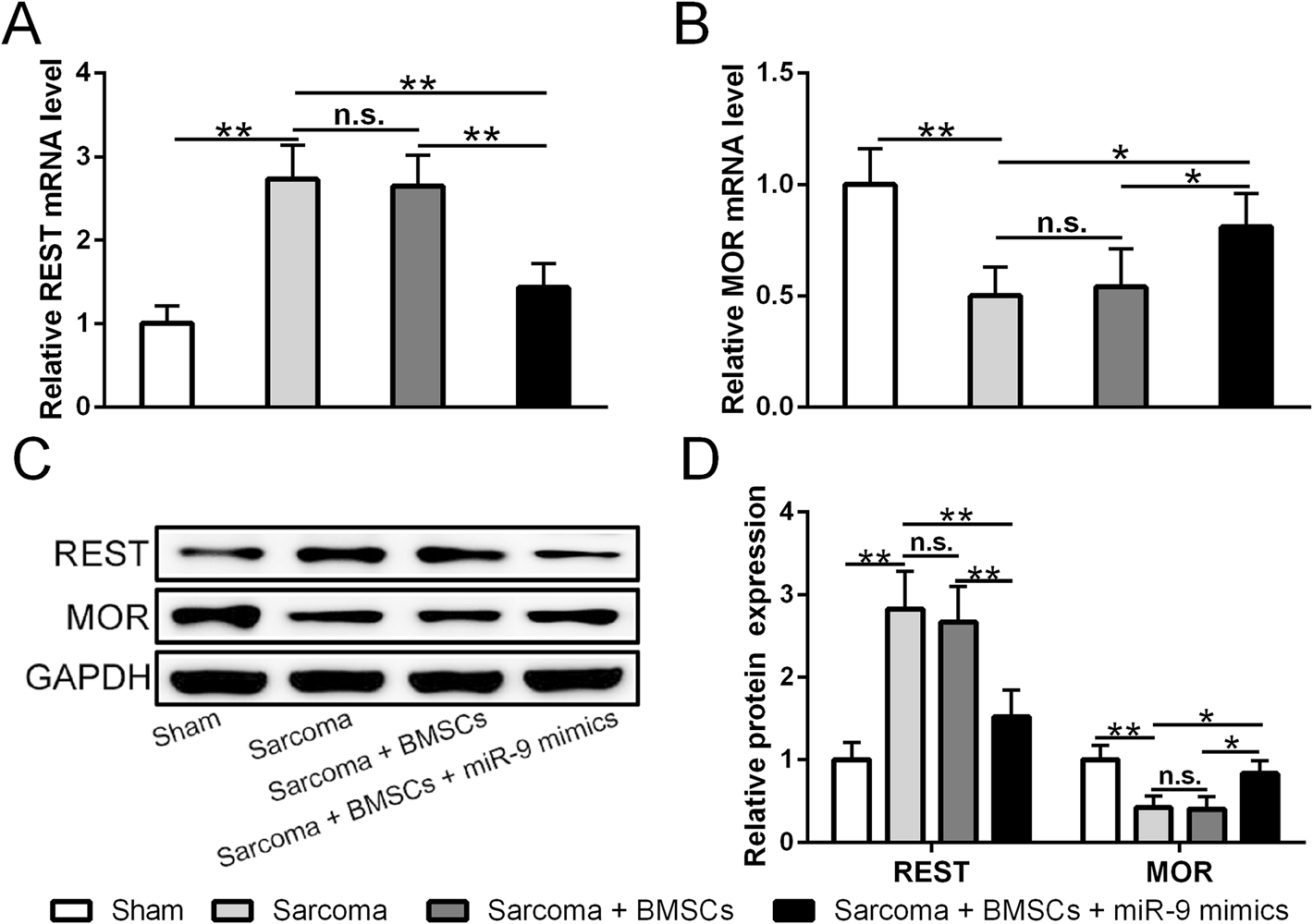
MiR-9-5p modified BMSCs regulated expressions of REST and MOR in ipsilateral dorsal root ganglion (DRG) of sarcoma-inoculated mice. **a** MiR-9-5p modified BMSCs reduced REST mRNA level at day 21 post operation. **b** MiR-9-5p modified BMSCs enhanced MOR mRNA level at day 21 post operation. **c** Accordingly, MiR-9-5p modified BMSCs reduced REST protein expression but enhanced MOR protein expression at 21 days post operation. **d** Quantification of protein expression presented in **c**. qRT-PCR was used to evaluate mRNA level and western blotting was used to evaluate protein expression. In each group, *n* = 6. Mean ± SD is used to present all data. *p < 0.05, **p < 0.01

**Fig. 7:**
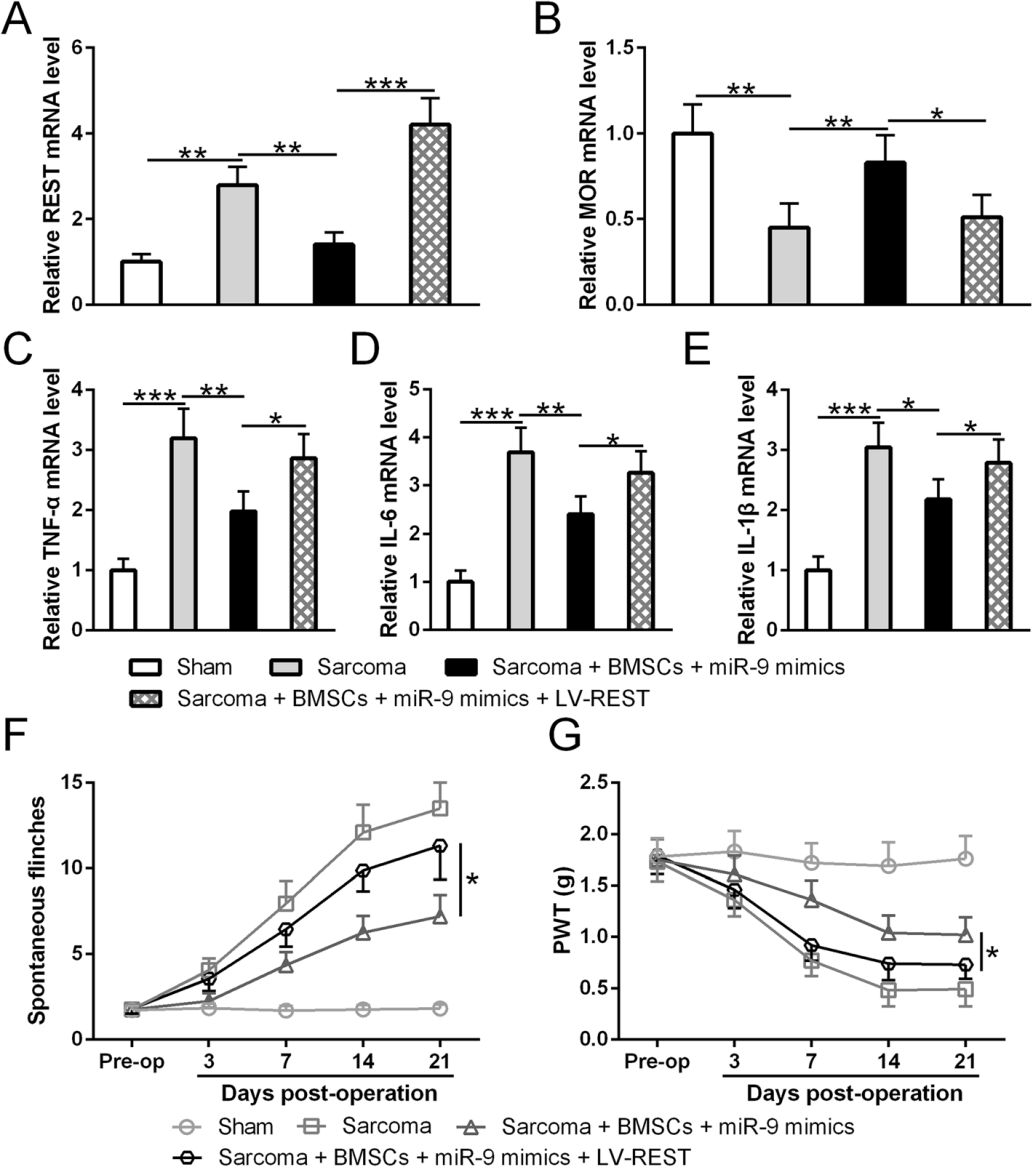
Overexpression of REST diminished antinociceptive effect of miR-9-5p modified BMSCs on the sarcoma-inoculated mice. **a** The expression efficacy of LV-REST was confirmed by increased REST mRNA level. **b** Overexpression of REST reduced MOR mRNA level at 21 day post operation. **c**–**e** Overexpression of REST diminished miR-9-5p modified BMSCs induced increase of TNF-α (**c**), IL-6 (**d**), and IL-1β (**e**) in ipsilateral dorsal root ganglion at day 21 post operation. In each group, *n* = 6. **f**, **g** Overexpression of REST diminished miR-9-5p modified BMSCs induced alleviation of spontaneous flinches (**f**) and paw withdrawal threshold (**g**) at 3, 7, 14, and 21 days after sarcoma cell inoculation. The proximal part of the catheter was sutured into the subcutaneous tissue to secure the catheter from being removed. Mice were grouped into four categories: control group, sham group; bone cancer pain group, and bone cancer pain + BMSC group which received 6 × 10^6^ cells/10 μL BMSC infusion every day started 2 days before murine sarcoma cell implantation till day 21 post operation. BMSC+miR-9 and LV-REST were delivered in parallel. Overexpression of REST diminished miR-9-5p modified BMSCs. In each group, *n* = 6. Mean ± SD is used to present all data. *p < 0.05, **p < 0.01, and ***p < 0.001

Since cytokines in the nervous system are the key players mediating pain sensation, we suspect that miR-9-5p modified BMSCs can regulate cytokine expressions in the central nervous system by directly targeting REST gene. We first examined the cytokine expression in the ipsilateral DRGs of sarcoma-inoculated mice. This suppressing capacity of BMSCs is significantly enhanced after modification by miR-9-5p (*p* < 0.01, Fig. 5a–f). We then tested the effect of BMSC infusion on the expression of REST gene and MOR gene. Consistent with our previous findings, BMSC infusion reduced REST expression but enhanced MOR expression in the DRGs of sarcoma-inoculated mice (Fig. 6a–d). Mir-9-5p modified BMSCs showed significantly stronger inhibiting effects than BMSCs alone (Fig. 6a–d). To further verify that BMSCs alleviate cancer pain through inhibiting REST expression, we overexpressed REST in the spinal cord. As expected, overexpression of REST significantly diminished BMSCs’ effect on REST and MOR expressions (Fig. 7a, b) as well as that on cytokine expressions (Fig. 7c–e). Accordingly, the overexpression of REST significantly diminished BMSCs’ alleviating effect on pain sensation (Fig. 7f, g). In summary, our results suggest that modifications mediated by miR-9-5p endow BMSCs more properties to alleviate cancer pain.

## Discussion

A growing body of studies have indicated that BMSCs have powerful analgesic effects in animal models of bone cancer pain [19]. However, the underlying molecular mechanisms remain unknown. In the present study, we manipulated BMSCs by transiently transfecting the cells with microRNA-5-9p mimic and then infused these cells into the spinal cord of mouse models of bone cancer pain. Our results demonstrate that microRNA-5-9P modified BMSCs significantly relieved pain-related behaviors by suppressing REST and cytokine expression and enhancing MOR expression (Figs. 4, 5, and 6). Therefore, our study suggests that the crosstalk among the molecular pathways of microRNAs, transcriptional factors, opiate receptors, and cytokines in the BMSCs might contribute to the pain alleviation mediated by BMSCs.

The bone cancer pain can be breakthrough pain which is severe spontaneous pain with unpredictable and acute occurrence [3]. The breakthrough pain is usually caused by bone remodeling process and can significantly disturb a patient’s functional status [3]. The pathophysiological changes in the central nervous system of the mouse model with bone cancer pain have been well studied [20]. The increase of cytokines is the consistent findings over the last decades that is believed to be the culprit leading to the persistent and resistant pain in pain sensation [21, 22]. Consistent with previous studies, as shown in Fig. 5, our results show that TNF-α, IL-6, and IL-1beta expression increased in our mouse model of bone cancer pain. In addition, our results show that cytokines can self-amplify through an unknown positive feedback loop in cultured cells (Fig. 2c–e). Interestingly, microRNA-5-9p overexpression in BMSCs can break the self-amplification loop of cytokines. Furthermore, microRNA-5-9p modified BMSCs can inhibit cytokine expression in the spinal cord of the mouse model with bone cancer pain. Taken together, our results show that the effect of microRNA-5-9p on cytokine expression in vitro is transferable to the in vivo condition. One possibility is that BMSCs proliferate in the central nervous system and replace those activated glia cells. Recent studies suggest that transplanted BMSCs can secret cytokine transforming growth factor beta (TGFbeta) which is a potent anti-inflammatory cytokine [23]. It will be informative to check whether microRNA-5-9p overexpression can modulate TGFbeta release in BMSCs in future studies.

The crosstalk among molecular networks related to pain sensation involves a complex regulation among transcriptional factors, opioids as well as its receptors, cytokines, and gene expression regulators like microRNAs. For decades, opioids remain to be the major prescribed medication in the management of cancer pain [9]. Generally, high dose of opioids is required to achieve modest pain control. However, escalating doses of opioids causes a variety of disabling adverse effects. In addition, 10–30% of patients with cancer pain cannot resolve pain even with high dose of opioids [24]. Recent studies have shown that aberrant regulation of MOR expression might contribute to the pathogenesis of persistent pain in cancer patients [25]. However, how MOR expression is regulated remains to be elucidated. REST is a well-known transcriptional repressor in the nervous system. REST has been reported to suppress MOR expression not only in cultured cells but also in animal models [14]. Furthermore, a recent study demonstrates that modulation of MOR by REST is involved in pain sensation in animal models [14]. In the present study, we show that microRNA-5-9p modulates MOR expression by directly targeting REST which subsequently reduced cytokine expression in BMSCs. Interestingly, our results also show that cytokine treatment in the cultured BMSCs can enhance REST but inhibit MOR expression that probably in turn leads to the increase of cytokine expression (Figs. 2 and 3). Therefore, our results suggest that microRNA-5-9p can modulate the crosstalk among transcriptional suppressor REST, MOR, and cytokines. Changes in molecular properties caused by microRNA-5-9p overexpression endow BMSCs’ robust capacity to relieve pain sensation. Our results suggest that genetically modified BMSCs can be a promising cell therapy for pain management.

## Conclusion

To summarize, our results show that microRNA-5-9p modified BMSCs can relieve bone cancer pain via modulating neuroinflammation in the central nervous system.

## Data Availability

Not applicable.
